# Forensic toxicology analysis of self-poisoning suicidal deaths in Tehran, Iran; trends between 2011-2015

**DOI:** 10.1186/s40199-017-0181-1

**Published:** 2017-06-13

**Authors:** Roya Kordrostami, Maryam Akhgari, Maryam Ameri, Masoud Ghadipasha, Kamran Aghakhani

**Affiliations:** 1 Forensic & Legal Medicine Department, Iran University of Medical Sciences, Tehran, Iran; 2 Legal Medicine Research Center, Legal Medicine Organization, Tehran, Iran

**Keywords:** Suicide, Self-poisoning, Forensic toxicology, Drugs, Poisons

## Abstract

**Background:**

Suicide ranks among the top ten causes of death in all age groups all over the world. There are many methods for committing suicide including self-poisoning, firearm and hanging. The aim of the present study was to provide an overview of self-poisoning related suicidal deaths with special focus on forensic toxicology analysis results in Tehran, Iran from 2011 to 2015.

**Methods:**

All suspicious cases with the the history of self-poisoning were investigated to define the cause and manner of death under the supervision of forensic medicine practitioners. Postmortem samples were analysed in forensic toxicology laboratory to confirm the presence of drugs in cadaver of suicidal cases. Drugs and poisons were analysed using thin layer chromatography, high performance liquid chromatography, gas chromatography/mass spectrometry, headspace gas chromatography and gas chromatography equipped with nitrogen phosphorus detector. Demographic data were collected from autopsy reports of all cases with confirmed self-poisoning suicidal cause of death.

**Results:**

Results showed that 674 cases of self-poisoning deaths were investigated during a five-year study period, of which 68.55% were male. The most often used suicide method was self-poisoning in young population. Phosphine gas liberated from aluminum phosphide tablets was the most toxic substance detected in postmortem samples (619 cases) followed by opioids, methamphetamine, organophosphates, cyanide and strychnine.

**Conclusion:**

In conclusion self-poisoning suicidal death was predominant in young male population in Tehran, Iran. It seems that free access to suicide means such as drugs and poisons should be restricted by national and health authorities.

**Trial registration:**

Not applicable.

## Background

Suicide is a complex, multifaceted problem and is categorized as one of the major causes of unnatural deaths in all age groups worldwide. Suicide is a public health issue that merits great attention and scrutiny [1]. Almost 800,000 suicidal deaths occur annually all over the world. This is equal to 16 per 100,000 people [2]. Asian countries account for about 60% of global suicides [3]. There is a 60% increase in suicide rates globally through the last 40 years. However suicide attempts occur up to 20 times more frequently than fatal and completed suicides [4]. The World Health Organization (WHO) estimates that the number of suicidal deaths will reach 1.5 million people in 2020 [5]. Suicide rate is growing in Iran. Iran ranked 91st in suicide all over the world in 1991, this rate reached 58th in 2003 [6]. Hassanian-Moghaddam et al. reported that Iran had shown the highest increase rate of suicidal deaths among Eastern Mediterranean Region and Islamic countries [7]. Intentional poisoning is the third method of suicide following hanging and self-burning in Iran [8]. Although non-medical use of licit and illicit drugs has increased substantially in recent years in Iran [9], studies examining the presence of drugs and poisons in postmortem samples of completed self-poisoning suicide are relatively scarce.

Kiadaliri et al. in a study conducted in Iran, investigated overall and social inequality in suicidal deaths across all provinces from 2006 to 2010 [10]. Saberi-zafaghandi et al. had shown that drug and narcotic overdose and also poisoning were the most usual suicide attempts in Iran [11]. The role of drugs and substances in violent suicide was discussed by Sheehan et al. [12]. Previous studies investigated the toxicology findings in suicides and the relation of ethanol and other drugs to hanging and poisoning [13]. In their 2 years cohort study of suicides, Galway et al. indicated that alcohol, licit and illicit drug use play a significant part in suicide in Northern Ireland [14]. The prevalence of non-medical use of psychoactive prescription drugs among adolescents was investigated to explore the role of these drugs in suicidal ideation by Juan et al. [15]. Chemical poisoning was defined as the fourth most common method of suicide in geriatric population in Turkey through 2009–2013 [16]. Suicide attempts and completed suicides in Iran from 1981 to 2007 was analysed by Shirazi et al. They analysed 54 published studies concerning suicide and concluded that drug poisoning was the most common method of attempting suicide in their study period [17]. Rate of completed self-poisoning suicides in each community can offer insights into the unique configuration of risk factors such as access to drugs and poisons. There is always a frequently asked question from jurisdiction authorities, whether drug poisoning was the cause of death or the person was under the influence of drugs or substances at the moment of suicide or other criminal offence. Although it is now broadly accepted that drugs and poisons have key role in suicidal act, very little is known about analytical toxicology results of postmortem samples obtained from self-poisoning completed suicide cases and there is few published research in this area in Iran. Therefore we undertook a retrospective analytical study to investigate toxicology findings in completed suicides referred to Legal Medicine Organization, Tehran, Iran to define the most prevalent drugs and poisons in postmortem samples of self-poisoning completed suicides in a five-year study period (2011–2015).

## Methods

### Case selection

In this cross-sectional analytical retrospective study all self-poisoning suicidal deaths referred to Legal Medicine Organization, Tehran, Iran were investigated from forensic toxicology and forensic medicine point of view with respect to demographic data, forensic toxicology analysis results, and finally cause and manner of death. Legal Medicine Organization is affiliated to the jurisdiction authority in Iran. All suspicious and unnatural deaths including homicide, suicide, drug poisoning related deaths and fire related deaths should be reported to Legal Medicine Organization for examination and to issue death certificate. Approximately 10,000–11,000 cases are referred to Legal Medicine Organization, Tehran, Iran annually to clarify the cause and manner of death. About 35% of these cases are referred to forensic toxicology department. All self-poisoning suicidal deaths during 2011–2015 were included in the present study. Other manners of suicidal deaths such as hanging, burning and firearm were excluded.

### Biological sample collection

Blood (10–15 mL) was drawn from femoral vein. 20 mL of urine sample was collected from bladder. Vitreous humor was gathered by inserting a syringe needle into each eye. Right lobe of liver (250 g) and bile were collected separately. Stomach content was inspected for ingested pills or liquids prior to death and removed from the stomach to be gathered in bottles.

### Forensic toxicology analysis

#### Sample preparation methods

Liver, stomach content, urine, blood and bile samples were subjected to the extraction process using liquid liquid extraction (LLE) method. Liver samples were homogenized (Heidolph homogenizer, DIAX 900). As all drugs and poisons don’t have the same chemical structure, pH adjustment was used to extract weak acidic drugs (barbiturates, phenytoin, valproic acid, acetaminophen and primidone) and drugs with basic structures (narcotic analgesics, benzodiazepines, antidepressants, amphetamine type stimulants and phenothiazines). It should be taken into account that many drugs of interest in forensic toxicology have a basic structure [18]. Careful adjustment of pH of the medium equal to isoelectric point of amphoteric drugs such as morphine is needed for efficient extraction using LLE method [19]. PH of the homogenized liver sample, bile and stomach content was set to acidic (pH = 2), basic (pH = 12) and neutral (pH = 7–9). Acid hydrolysis is needed for drug conjugates (metabolites) cleavage in urine sample. To perform acid hydrolysis the pH of urine sample was adjusted to 1–2 with hydrochloric acid and the sample was incubated at 60 **°**C for 3 h. For the extraction of opioids and basic drugs the pH of the medium was adjusted to 7–9 and 12 respectively. Aqueous media were extracted with chloroform:isopropanol (8:2 *v*/v). The resulting organic layer was separated and evaporated to dryness. Extraction products were solubilized in the minimum amount of methanol and prepared to be analysed by thin layer chromatography (TLC). Positive results obtained from TLC procedure were confirmed with more sensitive and specific analytical instruments such as high performance liquid chromatography (HPLC) (Knauer, Germany) equipped with diode array detector (DAD) (Knauer DAD 2700, Germany) and an Agilent GC/MS instrument (USA) consisting of a 7890 A GC and a 5975 C mass detector. Quantitative analysis of methanol and ethanol in blood and vitreous humor samples was performed using a headspace gas chromatography system (Agilent 6890 N, USA) equipped with flame ionization detector. Carboxyhemoglobine was analysed in blood samples using Cecil 9000 spectrophotometer. Postmortem samples of all cases were searched for methamphetamine and amphetamine using heptafluorobutyric acid (HFBA) as derivatization reagent and previously validated method for GC/MS instrumentation [20]. Cyanide was detected in blood, stomach content or liver samples using Prussian Blue test (a colorimetric and screening method) and polarography/voltammetry (Metrohm 797 analyser) technique as confirmatory test [21]. Phosphine gas (PH_3_), liberated from aluminum phosphide or zinc phosphide was analysed by silver nitrate (AgNO_3_) impregnated paper test according to the method described by Chugh et al. [22]. Positive results were confirmed by headspace gas chromatography with nitrogen phosphorous detector (HSGC/NPD) [23].

#### Decedents’ data collection

Data regarding self-poisoning suicidal deaths that were referred to Legal Medicine Organization, Tehran, Iran were collected from death certificates between March 20th, 2011 and March 21st, 2016. During the 5-year study period 55,210 deaths were investigated in Legal Medicine Organization, Tehran, Iran, of which, 19,412 (35.2%) were referred to forensic toxicology department. Self-poisoning suicidal deaths accounted for 674 cases and enrolled in the study. Other suicide methods such as hanging and firearm were excluded from the study. Death certificates of cases were reviewed and extracted data such as age, gender, marital status, employment status, forensic toxicology results for licit and illicit drugs, alcohols and poisons were entered into the designed questioners by a trained forensic toxicology specialist.

### Statistics

Statistical analysis was performed with SPSS software (Chicago, IL, USA) using Kolmogorov-Smirnov test, Mann–Whitney U test and Chi-square test. Categorical variables are shown with frequency and percentage (%). Mean ± standard deviation (SD) was used for continuous variables. *P* values < 0.05 and confidence interval (CI) which do not include odds ratio (OR) = 1 were considered to be statistically significant.

## Results

### Demographic characteristics

**Fig. 1:**
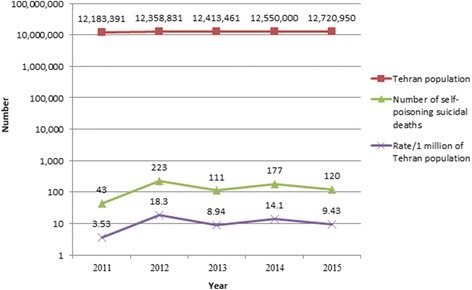
The number and rate of positive toxicology results in self-poisoning suicidal deaths per one million of population, Tehran, Iran, 2011–2015

**Fig. 2:**
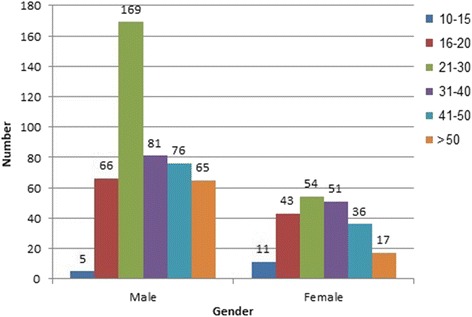
Distribution of positive toxicology results according to different age groups and gender in self-poisoning suicidal deaths, Tehran, Iran, 2011–2015

**Fig. 3:**
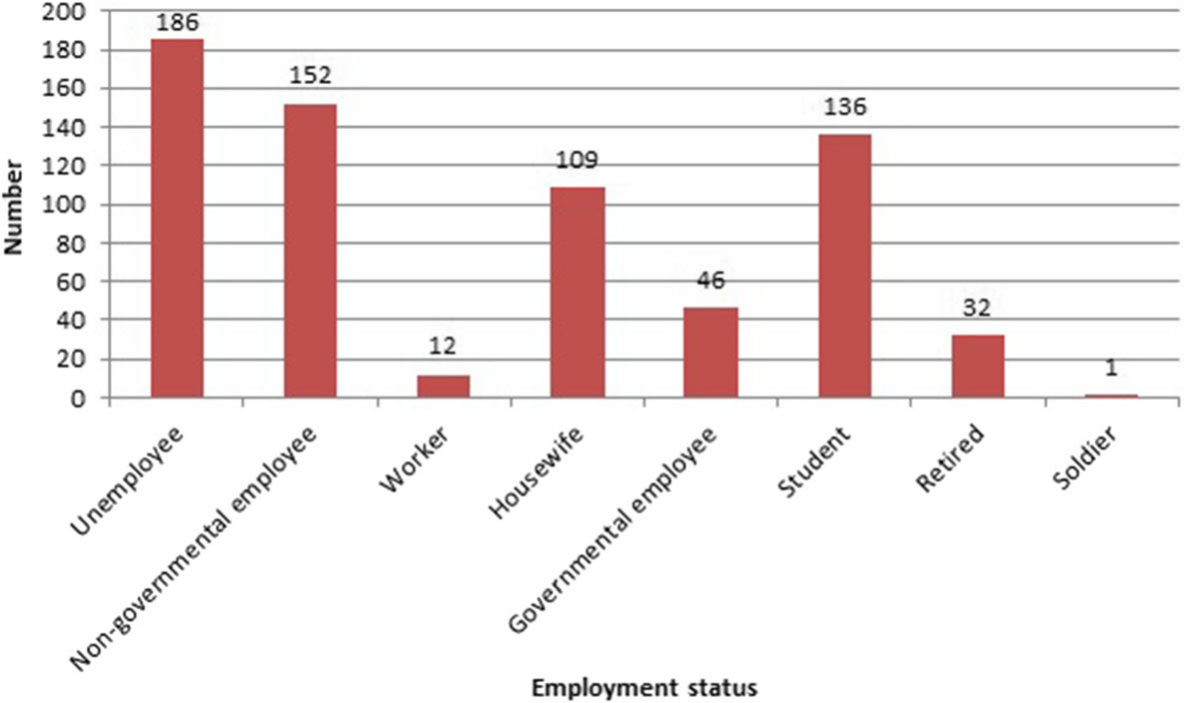
Employment status of self-poisoning suicidal death cases, Tehran, Iran, 2011–2015

In five-year study period 1667 suicide cases were investigated in Legal Medicine Organization, Tehran, Iran, of which, 674 cases (40.43%) showed positive results for drugs and poisons in postmortem samples obtained from cadavers as a result of self-poisoning. These comprised 462 (68.55%) males and 212 (31.45%) females. Thus male self-poisoning suicide death rate was about two times that of women. Figure 1 shows the number and rate of positive toxicology results per 1 million of population in each year in Tehran province, Iran. The rate for total self-poisoning suicidal deaths was determined as lowest in 2011 (3.53/1 million of population) and highest in 2012 (18.30/1 million of population). Mean age of the cases was 32.61 ± 13.7 (mean ± SD). Figure 2 shows the distribution of positive toxicology results according to different age groups and gender. There are significant differences in death rate across 6 age groups (*p* < 0.01)*.* In this respect self-poisoning deaths were more common in young male population (20–30 years old). A separate analysis examining self- poisoning showed that this suicide method was most common methods of suicide in younger people compared to older population (OR = 3.615, CI = 2.21–5.02, *p* < 0.001). The odds of intentional poisoning deaths were significantly higher in male subjects in comparison to females (OR = 2.75, CI = 2.2–3.43, *p* < 0.001). Most of the poisoning cases were not working; employment status showed that 27.6% (186 cases) were unemployed. Figure 3 shows the employment status of all self-poisoning suicidal cases. All of the cases were self-poisoned by oral, inhalation and injection routes. Suicidal poisoning was significantly more common in single subjects (360 cases) in comparison to married (313 cases) and divorced (1 cases) ones (*p* < 0.001). As for educational level, the highest rate of self-poisoning suicidal deaths was observed in subjects with high level of educational attainment (diploma/university degree) compared to low educational attainment level (primary/middle school). Table 1 shows the educational levels of cases in the study. Educational attainment appears to be an important factor in suicidal poisoning. Self-poisoning suicidal deaths were higher in high educational level subjects (OR = 3.38, CI = 2.47–4.62, *p* < 0.05). The seasons with the significant highest number of suicidal poisonings were spring (*n* = 190, 28.2%) and autumn (*n* = 168, 24.9%).

**Table 1:** Educational status of cases in self-poisoning suicidal deaths, Tehran, Iran, 2011–2015

Educational level	Number
Illiterate	19
Primary/middle school	132
Diploma	357
University students	136
University degree	30

### Forensic toxicology analysis results

Forensic toxicology analysis results had shown 766 positive results for drugs, alcohols and poisons. Ethanol was detected at >35 mg/dL concentration in blood and vitreous humor samples of 10 cases (1.48%) of drug poisoning suicidal deaths. Cases that showed positive ethanol results were all males (100%) in the age ranges of 21–40 years. Table 2 shows vitreous humor alcohol concentration in 10 cases.

**Table 2:** Vitreous humor alcohol levels (mg/dL) in ten cases of self-poisoning suicidal deaths, Tehran, Iran, 2011–2015

Vitreous humor ethanol level (mg/dL)	Number
<50	2
50–80	1
81–150	3
151–300	2
301–450	2

**Fig. 4:**
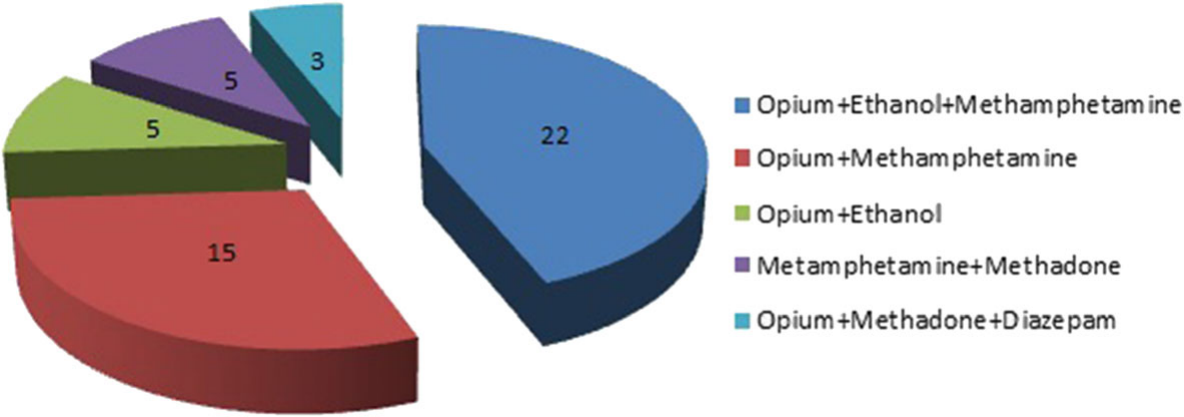
Pattern of polysubstance use related death cases in self-poisoning suicidal deaths, Tehran, Iran, 2011–2015

Table 3 shows drugs and poisons detected in postmortem samples. As expected, more than one drug was used by subjects to commit suicide. Figure 4 shows the pattern of poly drug use by 50 subjects in the present study. It should be noted that suicide victims had used alcohol with opium alkaloids, methadone, and benzodiazepines. The most prevalent toxic substance that was detected in postmortem samples was phosphine gas (619 cases) liberated from aluminum phosphide or zinc phosphide tablets or powders. Also organophosphates, organochlorine, cyanide and strychnine were detected in postmortem samples.

**Table 3:** Drugs and poisons detected in postmortem samples of cases in self-poisoning suicidal deaths, Tehran, Iran, 2011–2015

Drug category	Number
Opioids	
Opium alkaloids (morphine, codeine, thebaine, papaverine)	39
Methadone	8
Buprenorphine	1
Levorphanol	4
Methamphetamine	21
Ethanol	10
Benzodiazepines	
Diazepam	3
Oxazepam	2
Alprazolam	1
Tricyclic antidepressants	
Nortriptyline	3
Amitriptyline	1
Pesticides	
Aluminum phosphide (Phosphine gas)	619
Diazinon (dimpylate)	15
Azinphos methyl	3
Malathion	3
Chlorpyrifos	2
Endosulfan	2
Strychnine	10
Cyanide	17
Total	764

## Discussion

This study investigates self-poisoning related suicidal deaths in Tehran, Iran in a five-year study period. A key finding of the current study is the young age and male predominance of deliberate self-poisoning deaths in Tehran, Iran. Forensic specialists always request toxicological analysis in addition to the autopsy findings to better approach of suicidal deaths. The presence of drugs and poisons in postmortem specimens justify self-poisoning suicide or behavioral impairment under the influence of drugs. It is important to note that, in Iran, legal authorities demand an autopsy in suspicious or undetermined violent/injury deaths (poisoning, homicide, suicide) to determine the actual cause and manner of death.

The total number of self-poisoning suicidal deaths was 674 with the predominance of men. Throughout the years of the study, we observed a variation in completed suicide rates in Tehran from 3.53/1 million of Tehran population in 2011 to a maximum of 18.30/1 million of Tehran population in 2012. In other studies the rate of suicidal cases in Egypt was estimated to be 1.6–3.5/1 million populations [24], 10/1 million in Sudan [25] and 21/1 million in Jordan [26]. It should be noted that we couldn’t decide about suicidal poisoning rates based on forensic toxicology analysis results alone. Many factors influence this variation. Not all drugs and poisons have the same chemical structure. Volatile toxic substances easily evaporate from the body at room temperature. Moreover detoxification programs clear the toxins out of the body, thus lead to obtain negative results. Also some intentional poisoning deaths may be misdiagnosed as accidental poisoning.

In line with other studies, results showed that suicidal poisonings were more frequent in men than in women in all age groups except for 10–15 years group. Male to female ratio was 2.14, resembling the supremacy of males for self-poisoning suicidal deaths. The predominance of men in self-poisoning drug related deaths had been reported by other investigators [5, 21, 27, 28]. Males often choose more violent suicide methods that will assure to reach their desired aim [29–31]. However the results are in disagreement with previous scholars in that they suggested the highest number of suicide attempts in females [24, 32]. An easy explanation relies on the fact that the current study had only analysed completed self-poisoning deaths and the rate of nonfatal suicide attempts were not included in the present study.

As reported in previous studies the rate of completed suicide in adolescent boys (15–19 years) is higher in comparison to their female counterparts. Moreover girls choose less lethal methods and the rate of suicide attempts is higher in girls than boys [33]. Our results demonstrated the higher rate of suicidal poisoning in male adolescents too.

Self-poisoning suicidal deaths were highest in the 21–30 years age category. This result has also been observed by Rostami et al. in Kermanshah, Iran and Issa et al. in Saudi Arabia [1, 34]. Although other studies showed that participants attempting non-fatal self-poisoning were less than 25 years of age, suicidal intent was more prevalent among older decedents [35]. This result was seen in the present study too. However there are reports that indicate suicide rate is higher in older populations in developed countries [36]. There are many risk factors for suicidal ideation in older adults. One of the most important indicators of suicide in later life is hopelessness. As recorded by Stanley et al., each death by suicide in older population is matched by four attempts. Nonetheless this rate is one for 25 attempts in younger population [37].

As for occupation the highest rate of completed suicide was among unemployed subjects. The present study found evidence of association between employment status and self-poisoning suicidal deaths. Existing studies suggest a strong correlation between unemployment rates and suicide ideation and rates [38–40]. In contrast to the results of the present study, World Health Organization (WHO) reported that employment was unrelated to suicidal behaviors in 17 countries [41]. To our knowledge economic crisis and social stress are among contributing factors for suicidal ideation. A higher frequency of poisoning was seen in single cases that were in accordance with the results of the other investigations [24]. Controversial data regarding marital status in suicidal deaths was reported in previous studies [28]. A possible explanation for our results can be the feeling sense of responsibility for the family after getting married. Results of the present study showed that self-poisoning suicidal death rates were higher among those with higher levels of education. However Shojaei et al. [42] showed that hanging was the most frequent suicide method in subjects with lower educational levels.

Spring and autumn were the most troubling seasons regarding self-poisoning suicidal deaths. However other seasons were reported as the most seasons with suicidal deaths in previous studies [24, 43]. It seems possible to associate the high rate of suicidal poisoning in spring with the beginning of Persian New Year holidays, economic problems and stress for New Year ceremonies. Also weather condition changes with increasing hours of darkness in autumn that may be depression trigger and feel overwhelmed with sadness.

Illicit drugs were determined in some cases in polysubstance abuse pattern (7.4% of 674 cases). Whereas illicit drugs were detected in about 29% of suicidal cases in the study of Dias et al. [28]. Misuses of alcohol, illicit and licit drugs are key risk factors for suicidal ideation. WHO highlights that some risk factors such as harmful use of alcohol and substance use disorders are contributing elements for suicide ideation in all age groups [4]. Substance Abuse and Mental Health Services Administration suggested that pain relievers, psychotherapeutic agents, benzodiazepines, anxiolytics and hypnotics are among the most commonly drugs used in attempting suicide [44]. Young populations are more likely to engage in risky behaviors such as alcohol and illicit drug use [45]. It is estimated that there is a strong association between substance abuse and suicide in all age groups [12, 14]. Heroin users and opiate addicts face an elevated risk of suicide compared to the general public [46, 47]. There is always a question that is “why certain drugs are used before attempting suicide? The answer is that suicide ideation is accompanied by tremendous stress and substance use may help the decedent to escape from this situation [46].

Methamphetamine was detected in postmortem samples of 21 cases and in some cases in combination with opium alkaloids, ethanol and methadone. Cardiotoxicity is one of the major side effects of amphetamine type stimulants. Methamphetamine causes acute adverse cardiovascular effects and even death through excessive release of some neurotransmitters such as serotonin, dopamine and norepinephrine [48]. Also methamphetamine abuse is accompanied by delusion, hallucination and suicide attempt [49].

In the present study ten cases showed positive results for ethanol above 35 mg/dL in blood and vitreous humor samples. In accordance with other studies on the role of alcohol in suicidal deaths, ethanol positive cases where chiefly male in the young age range [34]. Demographic characteristics influence the association between alcohol use and suicide method [45]. Conner et al. stated that alcohol consumption is more probable in firearm or hanging suicide methods in comparison to self-poisoning suicidal method [50]. Even though alcohol use is not the only suicide risk factor, it acts by increasing disinhibition thoughts and behaviors. Therefore it can increase the likelihood of suicidal behavior occurrence [45]. Also it is reported in other studies that ethanol concentration above 50 mg/dL lead to higher suicide risk as a result of looking for courage and disinhibition in individuals when committing suicide [28]. Ethanol is one of the psychoactive substances listed as the most important drugs detected in postmortem analysis worldwide [34, 51–53]. As a result of increase in ethanol consumption in male population [54], ethanol was the most detected substance in a study conducted in Portugal and Sweden on suicidal deaths [13, 28]. However results of the present study showed that ethanol role in suicidal deaths was less than other studies. This may be explained by religious beliefs that have impressive effects on subject’s attitude towards alcohol use in Iran.

Determination of drugs (legal & illegal) and toxic substances plays an important role in death investigation of suicidal cases [34, 55]. Non-medical use of drugs has increased substantially in Iran. Per capita use of drugs in Iran is three times more than global standard [56]. Also Iran has the highest per capita use of opioids in the world [9]. One reason for this high amount of drug use is the free availability to drugs without prescription from legitimate pharmacy channels and through non-medical sources [57, 58]. Ease of availability is one of the important factors for choosing drugs and poisons as suicide means [35, 59]. The correlation between drug use and psychological distress is unclear and does not imply conclusion, in that which one causes the other [15]. Toxicology analysis results showed that 50 subjects were polysubstance abuser and the cause of death was determined as intentional poisoning. Drug use is one of the most ways of suicide in Iran [60]. Khabaronline reported that opium is the most common abused substance in Iran. But the tendency for amphetamine type stimulants is still high [61]. People with substance abuse disorders attempt suicide six times more than non-drug users. Also most drug addicts attempt suicide by overuse of drugs in an overdose manner [62].

Phosphine gas was the most prevalent toxic substance detected in the present study. Aluminum phosphide is classified as fumigant pesticide that is available in Iran in 3-g tablets forms with the brand names of Celphos, Phostoxin, Quickphos to protect rice and other grains. Aluminum phosphide is converted to phosphine following exposure to moisture or hydrochloric acid in stomach [59]. Ministry of Health and Medical Education, Iran banned this highly toxic and fast acting substance since 2007 [63]. In spite of the ban on its production and selling, aluminum phosphide tablets are freely available in herbal shops with low price. Nowadays it became one of the most popular substances used for self-poisoning in Iran [59]. As far as we know, there is no study in the literature demonstrating high prevalence of suicide with aluminum phosphide in other countries. Use of some toxic substances such as aluminum phosphide in intentional poisoning was reported in previous studies [59, 64–66]. The high incidence of aluminum phosphide self-poisoning related deaths (80.8% of total positive toxicology results) in the present study would be due to the aforementioned factors. In our previous study that was conducted in Tehran, Iran, 85% of fatal phosphine poisoning related deaths were in suicidal cases [64]. In accordance with the results of the present study pesticides were the most used toxic products in self-poisoning suicides [28]. Also there are many research articles addressing intentional poisoning with aluminum phosphide in Iran, India and Morocco [59, 65, 67].

The cause of death of 25 cases was intentional poisoning with organophosphates. Use of organophosphate compounds with suicidal intent is common in developing countries with agricultural base [59, 68, 69]. Previous studies confirmed the use of organophosphates as suicide means [70, 71].

Intentional cyanide poisoning was the manner of suicide in 17 cases (2.2%) in the present study. Cyanide was detected in postmortem samples with opium alkaloids in five cases. This finding highlights the role of substance abuse in suicidal ideation. Men used cyanide predominantly (15 cases). This result was confirmed by our previous study on cyanide poisoning related deaths in Tehran, Iran [21]. The higher rate of intentional cyanide poisoning in men may be due to the fact that men are more likely to use more violent suicide methods to complete suicide [72].

Deliberate self-poisoning with strychnine was the cause of death of ten cases in the present study. Strychnine is a toxic alkaloid obtained from the beans of the plant strychnos nux vomica that is used as rodenticide. Poisoning with strychnine is uncommon and if suitable intervention is missed it can be fatal. It causes accidental poisoning in children and even suicidal poisoning in adults due to ingestion or inhalation [73]. If it is ingested, strychnine causes asphyxia and convulsion. Death occurs due to the paralysis of the brain’s respiratory center [74]. Ease of availability has been cited as a key factor for choosing strychnine as a suicide mean.

## Conclusion

Self-poisoning suicidal death is a major public health problem. A much smaller body of work has examined forensic toxicology analysis results in detail in self-poisoning suicidal deaths. This study investigated self-poisoning suicidal deaths from forensic toxicology point of view. Self-poisoning suicidal deaths were more prevalent in young male population. However there are numerous studies regarding the management of self-poisoning suicidal cases in medical detoxification and clinical toxicology wards in hospitals. It should be taken into account that not all suicide attempts result in decedents’ death, therefore the pattern of drugs and poisons used as an aid in suicide would be different in clinical and forensic cases. Meanwhile the results of the present study will help to better tailor preventive efforts regarding restriction for the access to suicidal means such as drugs and poisons especially aluminum phosphide tablets and powders.
